# The effect of sample type and location on industrial workplace sink and hand dryer microbiomes

**DOI:** 10.1186/s12866-025-04054-9

**Published:** 2025-05-26

**Authors:** T. P. Thompson, C. J. Rice, E. Athanasakis, J. Mawhinney, B. F. Gilmore, P. Fitzgerald, T. Skvortsov, S. A. Kelly

**Affiliations:** 1 School of Pharmacy, Queen’s University Belfast, 97 Lisburn Road, Belfast, BT9 7BL UK; 2 Randox Laboratories, 30 Randalstown Rd, Antrim, BT41 4FL UK; 3 School of Medicine and Centre for Interventions in Infection, Inflammation, and Immunity (4i), University of Limerick, Limerick, Ireland

**Keywords:** Antimicrobial resistance, Environmental surveillance, Hand dryer, Microbiome, Sink, Workplace

## Abstract

One major issue in tackling antimicrobial resistance (AMR) is the ability to effectively track resistance spread in environments where surveillance is limited. Such environments include those experiencing high volumes of hand washing and drying from multiple users. This study characterised the microbial populations and antimicrobial resistomes of two different sample types from a pharmaceutical industrial site as part of an AMR environmental surveillance programme. Paired samples were collected from hand dryers and adjacent sinks in distinct sampling locations: from toilets adjacent to ‘wet’ labs, and locations associated with ‘dry’ activities. Microbial populations in hand dryers were significantly different to those of sinks, whereas there was no significant difference based on sample location. The opposite effect was observed for resistomes, where profiles differed significantly based on sample location, but not sample type. When both sample type and location were considered together, differences in microbiomes were driven primarily by hand dryer profiles from different locations. Analysis of metagenomically-assembled genomes revealed the presence of many poorly characterised organisms, and suggested no specific families predominated in terms of ARG carriage. This study emphasises the impact of human activities in determining the resistome of commonly used appliances, and the need for continued AMR surveillance programmes.

## Introduction

One major issue in tackling the global antimicrobial resistance (AMR) crisis in the ability to effectively track the emergence and spread of genetic determinants of resistance in environments where surveillance is limited [1]. Large AMR surveillance programmes, such as the Global Research on Antimicrobial Resistance (GRAM) project, have been set up to address these concerns [2].

The AMR crisis is driven largely by overuse of antimicrobials in humans and animals [3–5]. This can be exacerbated by climate change, continuing population growth and increasing globalisation and urbanisation [6, 7], with resistance and microbial spread intensified in areas of high human activity [8–10]. Increased surveillance in these hotspots could play an important role in stemming the proliferation of antibiotic resistance genes (ARGs) [11]. Surveillance efforts have been facilitated by the recent advances in DNA sequencing capabilities [12]. This has made AMR surveillance more economically feasible, enabling surveillance efforts to be increased in high-risk environments which had previously been inadequately resourced [13].

Environments which can facilitate microbial and AMR spread include those which experience high volumes of hand washing and drying from multiple users. Several studies have investigated the ARG profile, or resistome, of wastewater and handwashing facilities in hospital environments [14–15], including in patient-facing areas [17]. Indeed, numerous studies have correlated contaminated hand-washing sinks and infection outbreaks in hospital settings, in particular with carbapenemase-producing Enterobacteriaceae [18–20]. Further studies have highlighted the presence of antibiotic resistant bacterial strains in sink environments [21–23].

Other multi-user environments, including communal wash rooms and public toilet facilities, have been the focus of considerably less research, in particular for large-scale metagenomic analyses. A number of studies have investigated the impact of hand washing and hand dryer use in these environments, and their effects on aerosolization of bacteria and direct environmental and user contamination [24–26]. One study found the concentration of aerosols and bacteria in air increased in these environments with increasing user numbers, with a further increase noted during the hand drying process itself [24]. High numbers of antibiotic resistant bacteria were found in hand dryers, paper towels, and door handles in a number of these environments [25]. One study, assessing the effect of hand dryers in shopping malls on hand hygiene, found a higher mean bacterial count on hands dried using a hand dryer compared to wet hands. This was considerably lower for hands dried using filtered drying systems [26]. The microbiological implications of hand dryer use have been the subject of a number of recent studies and reviews, including comparison to paper towel use and the potential for environmental microbial dispersal. There did not appear to be solid consensus on the most sanitary approach; however, most studies favoured the use of paper towels [27, 28].

Fewer studies still have examined the resistomes of industrial and workplace handwashing and drying facilities. In fact, there is a relative scarcity of microbiome profiling of the workplace environment more broadly, with little emphasis on investigation of ARGs in these studies [29–34]. A number of studies have examined ARGs in an indoor work environment, although these have often employed PCR approaches to target a limited number of ARGs, as opposed to full shotgun metagenomic sequencing [35, 36]. Given the high concentration of human activity and potential for bacterial dispersal associated with these activities, industrial and workplace handwashing and drying facilities represent one potential area of concern for microbial spread and AMR emergence.

This study aims to characterise the taxonomic profile and antimicrobial resistomes of a series of paired sink and hand dryer samples from an industrial site at Randox Laboratories, as part of a post-COVID-19 era AMR environmental surveillance programme. Specifically, the study aims to employ high-throughput shotgun metagenomic sequencing using Next Generation Sequencing (NGS) approaches to compare population and resistance profiles between paired samples across various locations, to generate MAGs to determine the taxa responsible for carriage of resistance determinants, and to investigate the effect of sample location on microbiomes within a busy industrial workplace environment.

## Materials and methods

### Sampling and DNA extraction

Sampling locations comprised three separate toilet lanes (locations A, B, and C), located adjacent to laboratories involved in molecular and microbiology activities, and three locations from Engineering and Manufacturing (E&M) departments (locations E, F and G), associated with dry lab and office activities (Table 1). Samples were collected from the water reservoir area of hand dryers (ATC Premium Blade Hand Dryer) and from the U-bend of sink pipes. Samples were collected using regular tip, peel-pouch 4N6FLOQSwabs (Thermo Fisher, UK), and the tip of each swab was placed into 400 µL sterile TE buffer (Thermo Fisher, UK). Swabs were vortexed at 2000 rpm for 1 min and DNA was extracted using the MagMax Microbiome Ultra-Nucleic Acid Isolation Kit (Thermo Fisher, UK), according to the manufacturers protocol. Briefly, 800 µL of lysis buffer was added to bead tubes before vortexing. 200 µL of the resuspended sample was added to bead tubes and vortexed for 10 min to ensure full cell lysis of the samples. Tubes were centrifuged for 2 min at 14,000 x g. 450 µL of this sample was added to the respective well of a NUNC 96 deep well plate (Thermo Fisher, UK). 40 µL proteinase K was added to each sample and the extraction proceeded using the KingFisher Flex purification system (Thermo Fisher, UK). 520 µL of bead mix was added to individual sample wells after 20 min, before being placed back into the KingFisher. Samples were eluted in 200 µL of elution buffer (Thermo Fisher, UK) and stored at -20 ⁰C until required.

**Table 1:** Summary of sampling locations

Sample type		Sample location	
Hand dryer (HD)	Sink (S)	Location description	Location type
HDA	SA	Toilet Lane A	Lane
HDB	SB	Toilet Lane B	Lane
HDC	SC	Toilet Lane C	Lane
HDE	SE	Engineering department	E&M
HDF	SF	Manufacturing department location 1	E&M
HDG	SG	Manufacturing department location 2	E&M

### DNA library preparation and sequencing

DNA concentrations were normalised and library preparation was carried out using an Illumina DNA Prep kit (Illumina, USA), following standard protocols. Briefly, 30 µL of each sample was incubated with 11 µL of bead linked transposons and 11 µL of tagmentation buffer. The solution was mixed via pipetting and incubated at 55 °C for 15 min, followed by incubation at 10 °C for 10 min. Following this, 10 µL tagmentation stop buffer was added to the reaction mixture and mixed via pipetting. The plate was sealed and incubated at 37 °C for 15 min, followed by a 10 min incubation at 10 °C. The plate was placed onto a magnetic stand for 3 min and subjected to two ethanol washes. The beads were resuspended in a PCR master mix consisting of 22 µL enhanced PCR mix (Illumina, USA) and 22 µL nuclease free water (Thermo Fisher, UK). The solution was mixed via pipetting before being placed on a Veriti thermal cycler (Thermo Fisher, UK) with the following reaction conditions. Lid set to 100 °C, 68 °C for 3 min, 98 °C for 3 min and 5 cycles of 98 °C for 45 s, 62 °C for 30 s, 68 °C for 2 min, followed by a final extension at 68 °C for 1 min. Amplified libraries were cleaned and resuspended in 30 µL resuspension buffer (Illumina, USA) before each sample was pooled into a final volume of 100 µL. The DNA was denatured and loaded onto a NovaSeq-6000 using an SP flow cell (2 × 250 bp) and 1% PhiX control DNA.

### Antibiotic resistance gene (ARG) analysis and genome comparison

ARGs were identified via the Resistance Gene Identifier (RGI) v6.0.2, using the Comprehensive Antibiotic Resistance Database (CARD) [37]. Genecounts were performed for each ARG identified across all samples and normalised based on the number of reads for each sample.

### Metagenome assembly and genome binning

Fastq files were quality-checked using FastQC [38] and assembled into contigs using SPAdes v3.14.0 in --meta mode, using k-mer lengths 21, 33, and 55 [39]. Assembled contigs were processed for taxonomic classification using Kraken v2.0.8 [40]. Assembled contigs were built into a bowtie2 database file using the bowtie2-build command associated with Bowtie2 v2.4.2 [41]. Reads were aligned to this assembly using the bowtie2 command, and Samtools v1.3.17 [42] was used to index the resulting.bam file. Contig depths were summarised using JGI v2.15 [43] and genomes were binned using the metabat2 command associated with the Metabat2 v 2.2.15 program [44]. Completeness and contamination of the resulting metagenomically-assembled genomes (MAGs) were checked using CheckM v1.1.3 [45] and those with > 5% contamination or < 50% completeness were discarded. PhyloPhlAn v3.0.60 was used to assign taxonomy to the selected MAGs [46].

### Visualisation and statistical analysis of the obtained results

Data were visualised using R version 4.1.0 and the packages ggplot2 version 3.3.6 and pheatmap version 1.2.12, and ClustVis web tool [47]. Statistical analysis included Kruskal-Wallis test with Dunn’s multiple comparison post-hoc test for comparison between groups and PERMANOVA for the analysis of differences between taxonomic and ARG profiles of metagenomes.

## Results and discussion

### Sampling and sequencing metrics

DNA was extracted and sequenced from six paired sink and hand dryer microbiomes across various locations at an industrial site at Randox Laboratories, a major UK-based clinical diagnostics company. DNA extraction and sequencing was performed in-house using KingFisher Flex robotic station and Illumina NovaSeq-6000 instrument, respectively. Environmental metagenomic sequencing yielded 250 bp paired-end reads and a total of 129.7 GB of sequencing reads across all samples. FastQC analysed a total of 575,332,508 sequences, with none of these flagged as being poor quality. The mean GC content of sequences across all samples was 63%. DNA sequencing reads have been uploaded to the NCBI GenBank database and are accessible under the accession numbers SRR30815466-SRR30815477 inclusive.

Sampling locations comprised three separate toilet lanes located adjacent to laboratories involved in molecular and microbiology activities, and three locations from Engineering and Manufacturing (E&M) departments associated with dry lab and office activities, as part of a wider AMR surveillance programme in various locations across the site. This enabled comparison not only of sample type (sink vs. hand dryer), but also of sample location and activities typically associated with each location type. Locations A, B, and C are a mean distance of 14 m apart. Locations F and G are 1 m apart, with considerably greater separation between location E and the other two ‘dry’ locations at a mean distance of 110 m. Locations A, B, and C as a group, as well as having closer proximity to labs with different associated activities, are also considerably separated from locations E (mean distance 53 m), and F, and G (mean distance 109 m) (Table 2).

**Table 2:** Approximate distances (in metres) between sampling locations

	A	B	C	E	F	G
**A**	0	11	20	45	99	99
**B**	11	0	12	55	107	107
**C**	20	12	0	59	120	120
**E**	45	55	59	0	109	110
**F**	99	107	120	109	0	1
**G**	99	107	120	110	1	0

### Taxonomic breakdown and population diversity of sink and hand dryer microbiomes

Analysis of the metagenomic datasets was conducted using Kraken2 v2.0.8, with 83.04 ± 7.86% of reads classified to at least Phylum level. At Phylum level, Proteobacteria were most abundant across all samples (with the exception of HDF, where Actinobacteria were most abundant), constituting a mean abundance of 78.33 ± 23.41% of classified reads. This was followed by Actinobacteria, with a mean abundance of 19.60 ± 23.67%. A similar profile was observed across both hand dryer and sink samples, with a higher proportion of Proteobacteria in sink samples (84.50 ± 7.17%) compared to hand dryers (72.16 ± 32.60%).

**Fig. 1:**
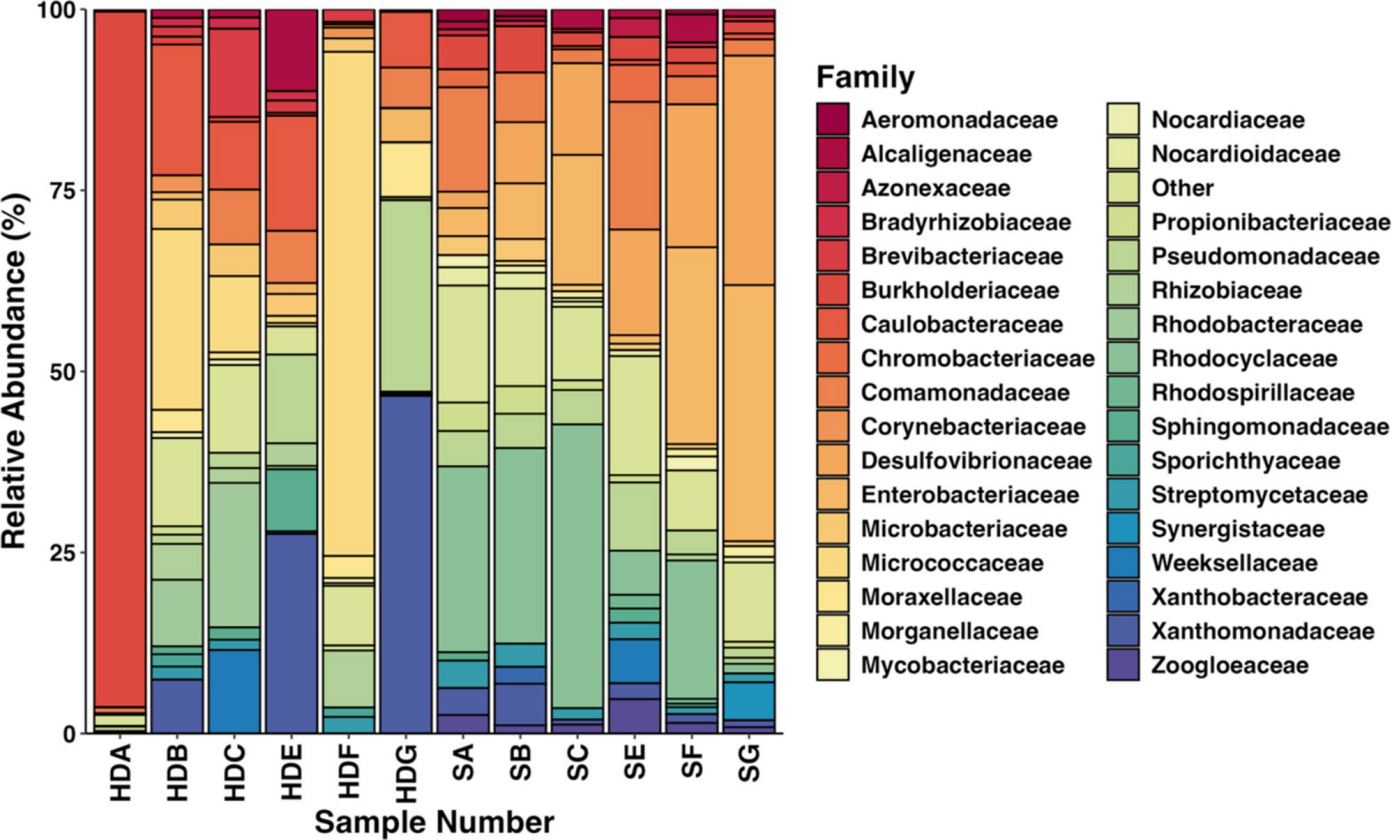
Taxonomic classification of sink and hand dryer metagenomes, showing relative abundance at Family level

A more nuanced picture of taxonomic differences between samples could be observed when profiles were examined at Family level (Fig. 1). In each sample, populations were dominated by a small number of bacterial families with high relative abundances, with a large number of families present at low relative abundances. In one particular sample, HDA, Burkholderiaceae accounted for 96.04% of all classified reads. Outside of HDA, relative abundance values for the most abundant family in each sample ranged from 17.63% (SE) to 69.61% (HDF). No bacterial families dominated across samples. This is illustrated by the fact that for nine of the top ten most abundant bacterial families, the standard deviation was higher than their mean relative abundance.

**Fig. 2:**
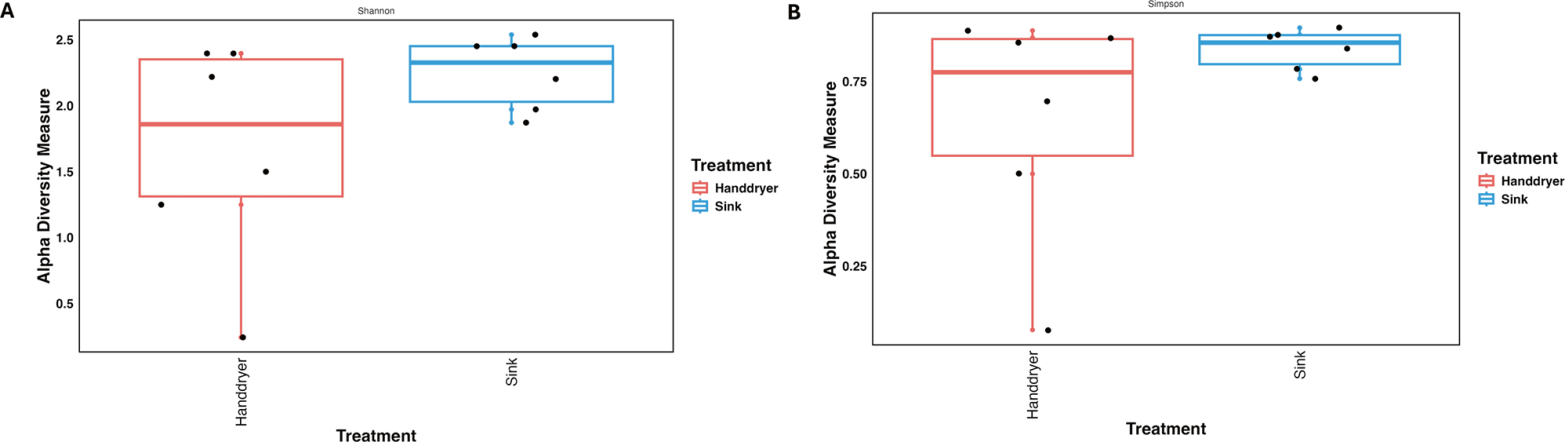
Alpha diversity analyses of sink and hand dryer microbiomes based on taxonomic OTUs at the Family level using both (**A**) Shannon, and (**B**) Simpson indices

Both measures of alpha diversity, Shannon and Simpson indices, showed no significant difference between hand dryers and sinks following Kruskal-Wallis tests (*p* = 0.1495 and 0.2623 for Shannon and Simpson indices, respectively). Shannon diversity measures were 1.67 and 2.25 for hand dryers and sinks, respectively, and Simpson diversity measures were 0.65 and 0.84, respectively (Fig. 2).

The differences in microbiome profile among samples and between sample groups is illustrated by PCoA plots following beta diversity analysis, using Bray-Curtis dissimilarity.

**Fig. 3:**
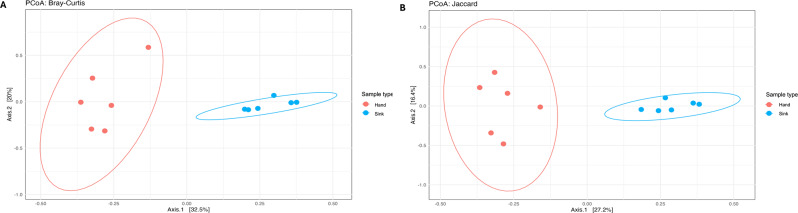
PCoA of beta diversity of sink and hand dryer microbiomes based on taxonomic OTUs at the Family level, showing profile separation based on sample type. PERMANOVA revealed statistically significant differences between community profiles at Family level between hand dryers and sinks, using both distance measurements (Bray-Curtis *p* = 0.002; Jaccard *p* = 0.004)

and Jaccard distance metrics (Fig. 3). These showed clear separation between the microbiome profiles of hand dryers and sinks. PERMANOVA revealed statistically significant differences between community profiles at Family level between hand dryers and sinks, using both distance measurements (Bray-Curtis *p* = 0.002; Jaccard *p* = 0.004). There was no significant difference between community profiles based on sample location (lane vs. E&M), using either distance measurement (Bray-Curtis *p* = 0.605; Jaccard *p* = 0.464).

Sample type had a significant impact on microbiome. Despite the greater distance between locations, community profiles from hand dryers in lane and factory locations were more similar to each other than to sinks in the same room. This data serves to highlight the vital role the characteristics a given ecological niche play in determining its microbiome. Previous literature investigating workplace and built environment microbiomes do not have consensus on this point, variously concluding that proximity to other surfaces does not influence community composition [48], with other work citing location as a key driver of built environment microbiome [29]. In the current study, this was significantly more important than proximity to paired appliances in the same room. This may be due to the environmental factors at play in each niche, which likely influences the types of bacteria that thrive therein. For example, the microenvironment of a hand dryer, such as temperature, humidity, and airflow, will be different to that of a sink U-bend, which is likely to have a greater concentration of soap residues or promote the selection of microorganisms co-inhibiting a mixed species biofilm formed under the conditions of intermittent liquid flow.

### AMR gene profiles from genomes assembled from hand dryer and sink metagenomes

**Fig. 4 A:**
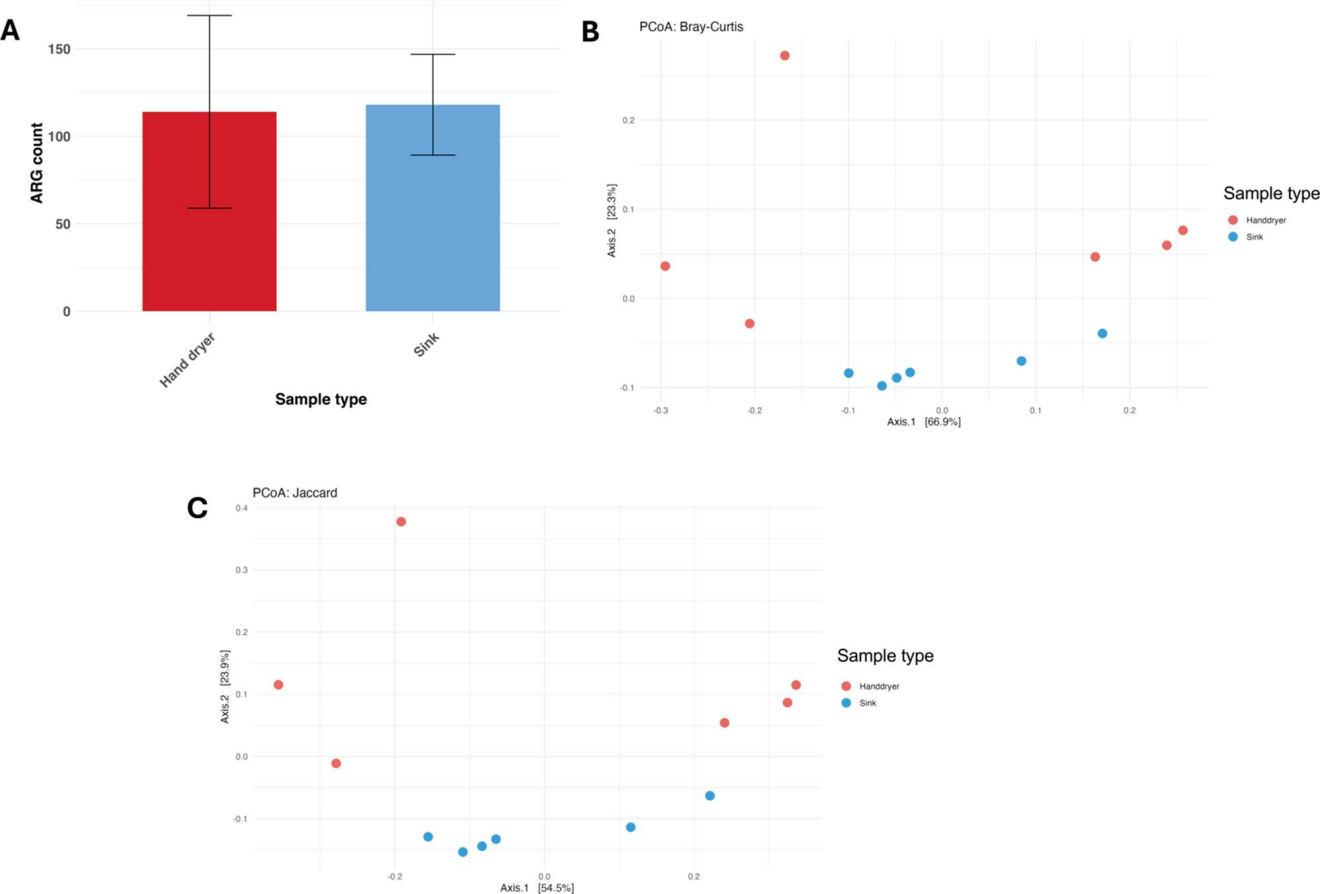
Mean ARG count in different sample types. Error bars show the standard deviation within each group. PCoA showing beta diversity of ARGs across sample types using **B.** Bray-Curtis and **C.** Jaccard distances

Antibiotic resistance genes (ARG) were identified from metagenomes using RGI tool and the CARD database, with gene counts used to determine resistome profiles. Across all samples, genes conferring resistance to fluoroquinolones were most abundant (*n* = 496), followed by tetracycline (427), disinfecting agents and antiseptics (242), and glycopeptides (235). When normalised by the number of reads, hand dryers had a mean ARG count of 114 ± 55 and sinks had a mean ARG count of 118 ± 29 (Fig. 4A). There was no significant difference between the numbers of ARGs in each sample, as determined by the Mann-Whitney U test (*p* = 0.9372).

There was also no significant difference in ARG profiles between hand dryers and sinks, as determined by PERMANOVA based on beta-diversity distances (Bray-Curtis *p* = 0.197; Jaccard *p* = 0.099) (Fig. 4B and C). However, there was significant a difference in resistome profiles between lane and E&M resistomes, when samples were grouped by location (i.e. hand dryers and sinks from lanes vs. hand dryers and sinks from E&M) (Bray-Curtis *p* = 0.003; Jaccard *p* = 0.005). This showed a significant link between sampling location and antimicrobial resistome, a feature which has received relatively little attention in microbiome analyses of the workplace environment. Investigation of these relationships is important in understanding the factors which determine proliferation and spread of AMR genetic determinants. Highlighting environments associated with gene patterns of concern may help in equipment design and risk management to reduce AMR emergence.

These results are noteworthy in that the opposite effect was observed in population profiles. Namely, population profiles were significantly different based on sample type (HD vs. sink) but not based on sample location (lane vs. E&M). These differences suggest that resistome profiles are not exclusively determined by taxonomic breakdown and abundance, and the ‘functionome’, including AMR genes, may also be influenced by other factors. This data suggests a given location may experience the same selective pressures for resistance genes, leading to more similar resistomes across different surface types within the same location.

These differences may also be due to human factors, such as the groups of users for the respective sinks and hand dryers and their normal activities. Lane toilets are primarily used by those undertaking wet lab molecular biology work, who tend not to frequently use toilets in the E&M departments. Similarly, E&M department toilets are used almost exclusively by staff working in these ‘dry’ environments, who tend not to use the toilets in lanes A, B, and C. The impact of human activities on microbiomes of the built environment is a well-described phenomenon [17, 49, 50]. In this study, we can see how the antimicrobial resistome of the built environment is also directly shaped by human activity.

**Fig. 5 A:**
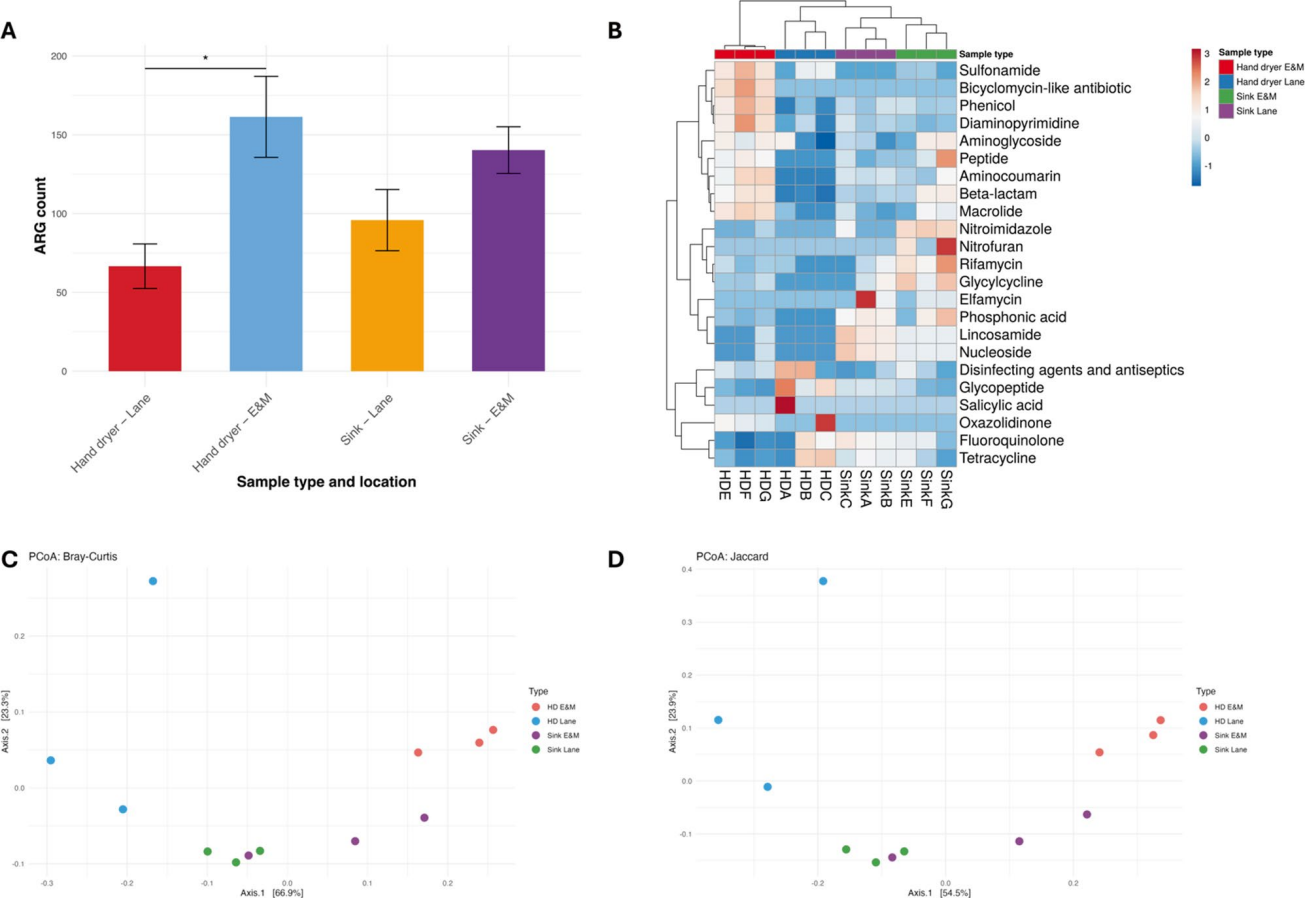
Mean ARG counts by both sample type (HD vs. sink) and location (Lane vs. E&M), showing significantly fewer ARGs in hand dryer samples from lane locations compared to hand dryers from E&M locations (*p* = 0.0134). **B.** Heatmap showing relative abundance of ARGs per sample. ARG profile relationships between samples are highlighted, with grouping seen among hand dryer samples and sink samples, respectively. Further groupings are observed within sample types by location, with hand dryer samples from E&M (red), separate to hand dryers from lanes (blue). Similarly, sink samples from E&M (green) groups separately to sink samples from lane (purple). PCoA based on ARG profiles beta diversity showing separation by location within sample types based on **C.** Bray-Curtis dissimilarity and **D.** Jaccard distances. Hand dryer samples from E&M (red), separate to hand dryers from lanes (blue). Similarly, sink samples from E&M (green) groups separately to sink samples from lane (purple)

Significant differences in resistome profiles were also observed when both sample type and location were considered, both in terms of overall ARG numbers and in ARG profiles. There was a significant difference in overall numbers of ARGs, as determined by the Kruskal-Wallis test (*p* = 0.0023). Dunn’s multiple comparisons post-hoc test determined that this difference was driven by the difference between hand dryer sample locations. The overall number of ARGs in hand dryers in lane samples (A-C) was significantly lower than hand dryers from E&M samples (E-F) (*p* = 0.0395) (Fig. 5A). This highlights, as also observed with community profiles, that human activities can have a significant effect on the number of ARGs recovered from a given environment. In this case, activities such as stringent hand and working surface disinfection using ethanol and other agents are likely to have had a considerable impact on the number of ARGs present. Figure 5B shows the relative abundance of ARGs by antibiotic class for each sample. A broad range of resistance determinants was present in both hand dryer and sink samples, including ARGs conferring resistance to many clinically relevant classes, such as beta-lactams, fluoroquinolones, macrolides, glycopeptides, and tetracyclines. The relationships between resistomes can be seen in the clustering tree at the top of Fig. 5B. This shows how ARGs cluster based on sample type (hand dryer vs. sink), and within this, subcluster based on sample location (Lane vs. E&M).

Groupings can also be visualised following PCoA analysis (Fig. 5C and D). Resistome profiles were significantly different when both sample type and sample location were considered, as determined by PERMANOVA and (Bray-Curtis *p* = 0.001; Jaccard *p* = 0.001).

**Fig. 6:**
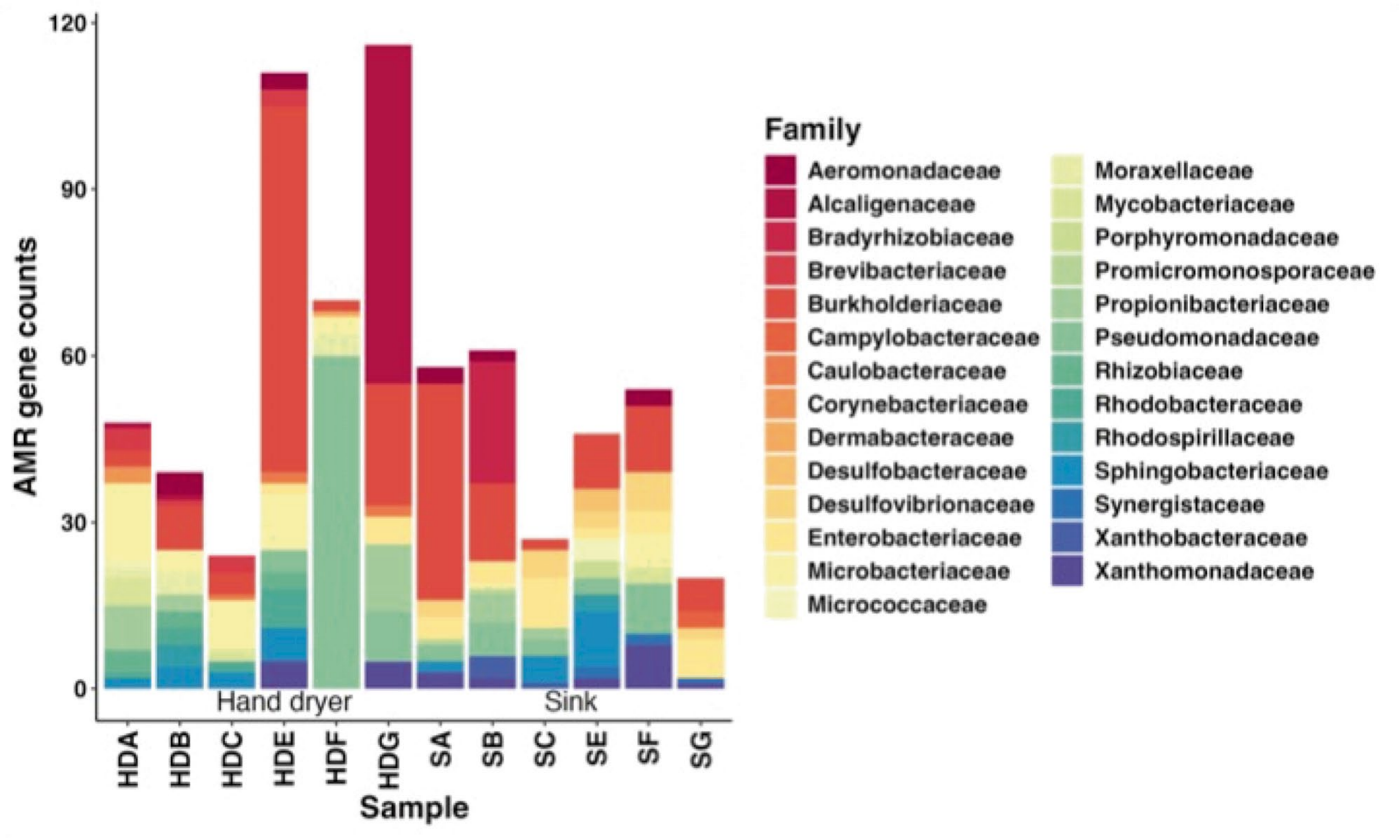
Total ARG counts identified in MAGs from each sample, showing the breakdown of ARG source by bacterial family following MAG identification

Following read-based analysis, MAGs were assembled in order to facilitate identification of the taxonomic source of resistance determinants (Fig. 6). This analysis can be useful in identifying specific taxa which play an outsized role in ARG carriage. Across all 12 samples, Burkholderiaceae accounted for the highest mean ARGs per sample (15.67), followed by Pseudomonadaceae (8.08), and Alcaligenaceae (5.25). However, as with taxonomic data, no bacterial families dominated across all samples, with high standard deviation values and high gene counts in certain samples skewing mean values. Of the MAGs generated only 63% on average were identified to Family level or lower taxonomic levels using PhyloPhlAn, suggesting a high degree of poorly characterised microorganisms were present in the samples investigated (Table 3).

**Table 3:** Metrics for metagenomically-assembled genomes (MAGs) from hand dryer and sink metagenomes

Sample	No. of MAGs generated^*^	No. of MAGs identified to Family level or lower^+^
HDA	43	27
HDB	29	19
HDC	27	13
HDE	27	21
HDF	8	6
HDG	13	11
Sink A	30	17
Sink B	25	15
Sink C	21	12
Sink E	38	20
Sink F	30	17
Sink G	24	14

^*^ Only MAGs with ≥50% completeness and ≤5% contamination, as per CheckM, were retained for analysis

^+^ As identified by PhyloPhlAn v.3.0.3

This study focused on the potential role of handwashing and drying facilities as reservoirs for microorganisms and ARGs in an industrial workplace setting. It highlights the advantages of in-house DNA extraction and sequencing technology and a conscientious AMR surveillance programme in the post COVID-19 era. Toilets represent another relevant niche, and surveillance could be expanded to include these environments. Recent research demonstrated how commercial toilets can emit energetic and rapidly spreading aerosol plumes, contributing to the dispersal of bacteria and viruses [51]. As such, air sampling in the immediate aftermath of toilet, sink, and hand dryer use could further bolster surveillance efforts in a bid to monitor ARG spread within workplace sanitation facilities.

## Conclusions

In this study, proximity between samples did not appear to be the most significant factor in determining microbial population profiles. It was anticipated that human activities between paired samples, namely using a sink for handwashing and the corresponding hand dryer to dry hands immediately after, may have resulted in a similar microbiome between paired samples. However, this did not appear to be the case. This work serves to highlight the importance of sample type in determining microbiome within the workplace, with hand dryers having a more similar profile to other hand dryers than to sinks in the same room. Furthermore, the impact of sample type on antimicrobial resistomes was explored. In this case, resistomes differed significantly between different locations within the industrial site, possibly due to shared selective pressures within a given location. This data could be important in informing design of handwashing and drying facilities, given their apparent role in dispersal of microorganisms and the ARGs contained therein, by identifying the relative abundance of potential pathogens and resistance determinants in different sample types and locations.

## Data Availability

The datasets generated and/or analysed during the current study are available in the NCBI GenBank repository and are accessible under the accession numbers SRR30815466-SRR30815477 inclusive.

## Abbreviations

AMR: Antimicrobial resistance
ARG: Antibiotic resistance gene
CARD: Comprehensive Antibiotic Resistance Databse
E&M: Engineering and manufacturing
GRAM: Global Research on Antimicrobial Resistance
HD: Hand dryer
MAG: Metagenomically-assembled genome
NGS: Next generation sequencing
RGI: Resistance Gene Identifier
